# Simplivariate Models: Ideas and First Examples

**DOI:** 10.1371/journal.pone.0003259

**Published:** 2008-09-23

**Authors:** Jos A. Hageman, Margriet M. W. B. Hendriks, Johan A. Westerhuis, Mariët J. van der Werf, Ruud Berger, Age K. Smilde

**Affiliations:** 1 Biosystems Data Analysis, Universiteit van Amsterdam, Amsterdam, The Netherlands; 2 ABC Metabolomics Centre, Lab. Metabolic and Endocrine Diseases, Wilhelmina Children's Hospital, Utrecht, The Netherlands; 3 TNO Quality of Life, Zeist, The Netherlands; Center for Genomic Regulation, Spain

## Abstract

One of the new expanding areas in functional genomics is metabolomics: measuring the metabolome of an organism. Data being generated in metabolomics studies are very diverse in nature depending on the design underlying the experiment. Traditionally, variation in measurements is conceptually broken down in systematic variation and noise where the latter contains, e.g. technical variation. There is increasing evidence that this distinction does not hold (or is too simple) for metabolomics data. A more useful distinction is in terms of informative and non-informative variation where informative relates to the problem being studied. In most common methods for analyzing metabolomics (or any other high-dimensional x-omics) data this distinction is ignored thereby severely hampering the results of the analysis. This leads to poorly interpretable models and may even obscure the relevant biological information. We developed a framework from first data analysis principles by explicitly formulating the problem of analyzing metabolomics data in terms of informative and non-informative parts. This framework allows for flexible interactions with the biologists involved in formulating prior knowledge of underlying structures. The basic idea is that the informative parts of the complex metabolomics data are approximated by simple components with a biological meaning, e.g. in terms of metabolic pathways or their regulation. Hence, we termed the framework ‘simplivariate models’ which constitutes a new way of looking at metabolomics data. The framework is given in its full generality and exemplified with two methods, IDR analysis and plaid modeling, that fit into the framework. Using this strategy of ‘divide and conquer’, we show that meaningful simplivariate models can be obtained using a real-life microbial metabolomics data set. For instance, one of the simple components contained all the measured intermediates of the Krebs cycle of *E. coli*. Moreover, these simplivariate models were able to uncover regulatory mechanisms present in the phenylalanine biosynthesis route of *E. coli*.

## Introduction

Modern instrumental methods have been generating a significant advancement in biology research. Especially in the field of functional genomics, transcriptomics and proteomics measurements have provided fundamental insight in many biological processes. The missing link between these measurements and the phenotype is called metabolomics [1]. This new field concerns the measurement of small biomolecules in body fluids, cells, tissues, etc. The type of data being generated in metabolomics studies is characterized by a very broad acquisition of semi-quantitative data of a large number of metabolites [1]–[4]. This results in data sets of a very complex nature. Not only are these data sets high-dimensional, they also exhibit mixtures of types of variation introduced by the specific experimental setup [5].

Traditionally, a set of measurements is analyzed by postulating a model describing systematic variation and assuming the left-overs (residuals) as being random. Due to the complexity of metabolomics data, this concept breaks down. There are many sources of variation in the data *non-informative* for the underlying biological question. An example of this type of variation are metabolites which are not under tight regulatory control and are thus allowed to vary almost independently across the experiments [6]. Such non-informative variation affects the data in a structured way and infiltrates the systematic or modeled part of the data. This results in poor interpretability and the failure to unearth subtle *informative* variation. In this paper, we propose a new conceptual framework for analyzing metabolomics data based on the idea to separate informative from non-informative variation. The informative variation should describe the systematic biological variation in relevant metabolites induced by underlying biological phenomena. What we are ultimately aiming for is to discover these biological phenomena.

Our assumption is that the studied biological phenomena are not represented by all measured metabolites, but that simple structures (subsets of related metabolites) in (parts of) the data exist, each simple structure or component describing an underlying biological phenomenon. In the development of our discovery tool we are aiming for a method that fulfills the following requirements: i) being able to identify simple structures, in which just a limited number of metabolites are represented by the structure; ii) representing each simple structure by a model, the type of model depending on the data collected and driven by *a priori* biological knowledge; iii) assuming that a (large) part of the data will most probably not be informative. The last assumption is reasonable given the holistic nature of metabolomics, where the aim is to measure all metabolites present.

**Figure 1:**
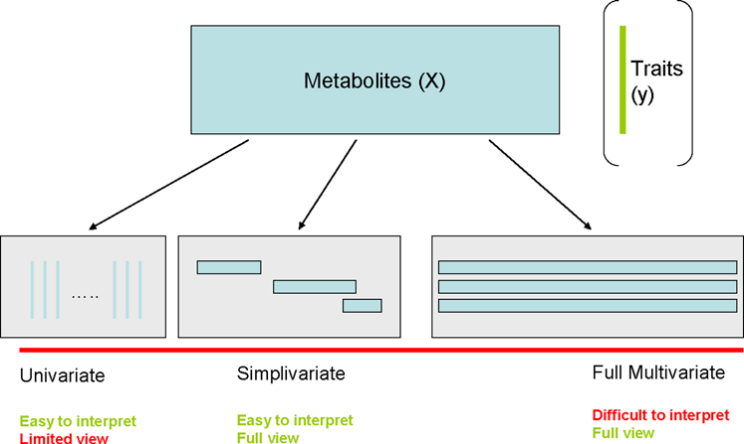
Graphical representation of the three different approaches to the analysis of multivariate data. From left to right: the univariate, simplivariate and multivariate approach.

We have called this new approach *simplivariate* models since they are in-between univariate and multivariate models and use simple building blocks (see Figure 1). Univariate models look at one-metabolite-at-a-time; they are easy to interpret but lack an overall view on the data since no correlations between metabolite values are used. On the other extreme are multivariate models; they provide a full view but often lack good interpretation especially in high-dimensional data cases. Simplivariate models try to have the best of both worlds: simplicity, comprehensiveness and correlation.

Although the simplivariate framework is general and can be used in exploratory analysis, regression analysis and discriminant analysis, in this paper we will focus on explorative methods. Usually in exploratory data analysis for metabolomics data, use is made of either of two types of techniques: projection (dimension reduction) methods or clustering methods. The first type of techniques (with Principal Components Analysis (PCA) as an example) searches for structures consisting of highly co-varying metabolites to construct new representations of the data [7]. Clustering techniques can roughly be divided into two categories: hierarchical clustering (based on linking objects or variables on dissimilarity measures), leading to a set of nested clusterings, and partitioning algorithms, where the result is just one partitioning, and a model is defined to represent the clusters. Both types of techniques do not fulfill the criteria i) to iii) of simplivariate models explained above, e.g., both PCA and hierarchical clustering do not look for components using a limited set of metabolites.

First, the simplivariate modeling framework will be presented in its full generality. Next, two techniques that fit into that framework will be discussed using real-life metabolomics data. Finally, shortcomings of these methods will be discussed and suggestions of improvement will be given.

## Materials and Methods

### Simplivariate models

A flexible framework is built by defining a *simplivariate* model that describes the partitioning of a data matrix **X** (*I* objects (e.g. experiments)×*J* variables (e.g. metabolites)) in components containing subsets of related variables (e.g. metabolites):
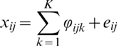
(1)


In which every element *x_ij_* of matrix **X** can be written as a sum of contributions from different components. These components *ϕ_ijk_* describe the *informative* parts of the data and can be very diverse in nature. The variation of *x_ij_* that is not included in factors *ϕ_ijk_*- *non-informative variation* - is indicated by *e_ij_*. Although the symbol *e_ij_* is commonly used to indicate random variation, it has a very different meaning here. The non-informative part is certainly non-random in the strict senses of randomness. To introduce the concept of simplicity not all variables are included in the factors *ϕ_ijk_*.
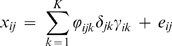
(2)


Here *δ_jk_* indicates the presence of variable *j* in component *k* and *γ_ik_* indicates the presence of an object *i* in component *k* (δ_jk_ = 1 if variable *j* is present in group *k*, 0 otherwise and γ_ik_ = 1 if object *i* is present in group *k*, 0 otherwise).

For simplicity we have used the same symbol *ϕ_ijk_* in equations (1) and (2), but their difference is clear from those equations.

When decomposing **X** into simple components, the idea is that interpretation will be easier, since not all original variables are included in those components. Only variables that are closely related will be used. In the case of metabolomics data, metabolites that are functionally related (e.g. part of the same pathway) may form a simple model.

### Simple structures

The components *ϕ_ijk_* can be very diverse in nature, and represent the relations between objects and variables in each of the subsets. Three examples of such component *ϕ_ijk_* are:
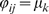
(3)representing simple component *k* by a constant. If this would be an exhaustive partitioning of all variables and objects this would resemble two-mode clustering [8]. Another simple model is
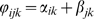
(4)which is a purely additive model for simple component *k*, that resembles a two-way ANOVA decomposition of a data matrix [8]. The next model to consider is
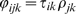
(5)which is a purely multiplicative simple component *k*, equivalent to a rank-one component PCA decomposition of a data matrix.

Combinations of representations Eq. 4 and 5 are also possible resulting in mixed models:
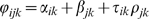
(6)The choice for one of these types of models should be based on information on the structure of the data and on *a priori* biological knowledge.

In equation (2) *δ_jk_* and *γ_ik_* indicate the presence of element *ϕ_ijk_* in factor *k*. For illustrative purposes, for the moment we will assume that all objects are present in every factor *k*, so γ_ik_ is always 1:
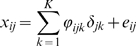
(7)


### Influence of preprocessing

The type of preprocessing applied to the data is influencing the outcome of an analysis [5], [9]. In the case of only searching for structures in the variables (so all objects are a member of all substructures, as is the case in for instance PCA), it is well-known [9] that the mixed models as mentioned in equation (6) can be treated as pure multiplicative models by first removing any sample or variable means by column or row centering. Apart from centring the data, also scaling can be applied to assure that less abundant metabolites (variables) have the same *a priori* chance to be important in the final model as more abundant metabolites. In our case, we do not partition in the sample direction. Hence, centering across the samples and scaling each variable to standard deviation one seems reasonable.

### Existing algorithms for simple models

There are several algorithms described in literature that can create simple models according to our definition in the previous sections. In this paper, we have chosen two algorithms, both representing both the multiplicative and additive model classes. In the following section, a short explanation of both methods will be given.

### Interpretable dimension reduction (IDR)

IDR [10] uses the PCA solution as starting point for creating simple models. By reducing and summarizing the number of non-zero elements of the loading vector, the loadings are simpler to interpret. IDR uses two constraints for obtaining simpler loadings of which the homogeneity constraint is used and discussed in this paper. This homogeneity constraint is applied to a loading that is obtained by PCA. Each loading value is rounded off to the nearest ±1. To increase the interpretability, zeros are introduced into the loadings, starting by replacing the absolute smallest loading values with zeros and continuing until the largest loading value is left over. Modified loadings are normalized. Each time after introducing another zero in the loadings, the angle to the original loadings is determined. The optimal number of inserted zeros is given by the lowest angle to the original variables and this one will be chosen. This method can either be used on a complete set of (PCA) loadings or in an iterative way simplifying one loading at a time. We use this method in a iterative way, deflating one simple component before starting with the next one. Step 1 to 8 of IDR with the homogeneity is as follows:

1. Set the *k* values of PCA loading vector *α* to 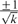, matching the sign with the original value.
2. Look for the absolute lowest non zero value of *α*, and set it to zero.
3. Calculate the inner product the original loadings vector *α* and the simplified *α*.
4. Convert the inner product to an angle with the inverse cosine.
5. Repeat steps 2–5 until only the largest absolute value is left over.
6. The simplified *α* that has the lowest angle is the optimal new IDR component.
7. Calculate scores (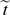) with optimal IDR component (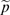): 
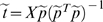
 Subtract the IDR component from the original data: 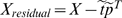

8. Repeat this procedure of all IDR components.

The final IDR model has the form:
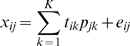
(8)Here *t_ik_* are the scores and *p_jk_* the loadings originating from PCA for component *k*. Many values of *p*_jk_ are zero. This can be made explicit by writing
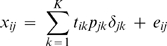
(9)where the symbol *δ_jk_* is the same as before and the nonzero values of *p_jk_* are either 1 or −1. Clearly, eq (9) is a special case of eqns (2) and (5) showing that IDR fits into the simplivariate framework.

### Plaid models

Plaid [11]–[13] is a form of two mode clustering that allows for overlapping clusters. By iteratively searching the data, plaid tries to find patches in the data that can be modeled by an ANOVA[7] decomposition. Objects or variables can be in more than one cluster or in no cluster at all. Plaid has originally been devised for micro-array data, but can be extended to other types of data.

The plaid model consists of a series of additive layers intended to capture the underlying structure of matrix **X**. The plaid model also includes the possibility of a background layer containing all variables and objects. Plaid models each cluster with standard 2-way Anova decomposition for each layer *k*:
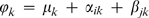
(10)where a *μ*_k_ is introduced to serve as a general mean (model (4) is essentially the same as model (10) [8]). This gives Eq. 11: the decomposition of matrix **X** into *K*+*1* plaid models assuming that all samples contribute to the plaid (as before):
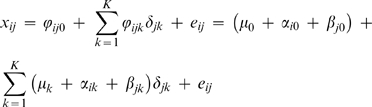
(11)


Here, *ϕ_ijk_* is the plaid contribution for element x*_ij_* from plaid model *k* and *ϕ_ij_*_0_ is the background layer model for entire the entire data matrix **X** (*I*×*J*). It can be seen that Eq. (11) is a special case of Eq. **(7)**. The background layer is especially important when dealing with micro-array data and can be used to model the background signal. This layer will be omitted from our analysis, because it has no meaning for metabolomics data. Instead the proper preprocessing will be used to correct for offsets and scale differences. An algorithmic overview of the plaid algorithm is shown below:

1. Choose starting values for 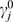 and 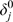 (indicating cluster membership)
2. Update layer effects using plaid cluster estimate *X_k_* using ANOVA decomposition. s indicates iteration number.
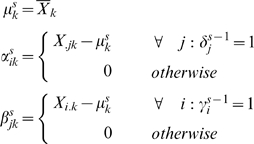

3. Update cluster membership
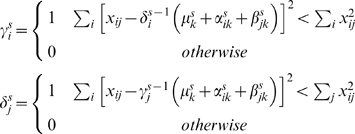

4. repeat step 2–3 for s iterations
5. Compute final layer effects as in step 2
6. Prune plaid cluster to remove ill fitting metabolites.
7. Test *X_k_* for significance, stop procedure if *X_k_* is not significant otherwise accept
8. Subtract *X_k_* from X
9. Apply backfitting for each obtained plaid cluster
10. Apply pruning to remove ill fitting metabolites and continue at step 2

The above algorithm is the original Plaid algorithm. We used it with some adaptations to our circumstances:

- we did not apply significance testing but selected 6 plaids for illustration.
- we applied a one step backfitting procedure
- we did used γ_j_ = 1 throughout and, hence, did not have to optimize those values.

When residuals of selected metabolites after the plaid fit are larger than the prune fraction (0.70, see Table 1), metabolites will be excluded from that plaid cluster. This mechanism ensures small and tight clusters in which the feature of the plaid cluster is clear in all members of the plaid cluster [12].

**Table 1:** Settings for the plaid algorithm.

Setting	Value
Maximum iterations	50
Number of permutation in significant testing	25
Backfitting	one step
Maximum number of layers	6
Prunefraction*	0.7

* Minimum of proportional reduction in residual sum of squares required for cluster membership.

### Background of the dataset

*E. coli* NST 74, a phenylalanine overproducing strain and *E. coli* W3110, a wild type strain were grown in batch fermentations at 30°C in a Bioflow II (New Brunswick Scientific) bioreactor as previously described [14]. In short, samples were grown on MMT12 medium with glucose as carbon source, a constant pH and a constant oxygen tension of 30%. Samples were taken at 16, 24, 40 and 48 hours and analyzed by GC-MS and LC-MS. Peaks related to the substrates used for growth (glucose and succinate) were removed from the data. Deliberate variations in the default protocol resulted in the experimental design that can be found in [14]. The resulting data set consisted of 28 measurements and 188 metabolites. Extensive details on experimental setup, GC-MS and LC-MS analysis and subsequent preprocessing can be found in [14].

Plaid and IDR were programmed in Matlab 7.1 [15] and are available on the internet at http://www.bdagroup.nl/downloads/bda_downloads.html. All computations were performed on an Intel Xeon 3.4 GHz computer with 3.25 GB of memory.

## Results and Discussion

Metabolomics data is highly dynamic in range. Metabolites can have very different and very large concentration ranges. Some metabolites will be zero since their concentrations will be too low to detect under some experimental conditions. This indicates that metabolomics data is not pure multiplicative in nature and can benefit from removing column means.

**Figure 2:**
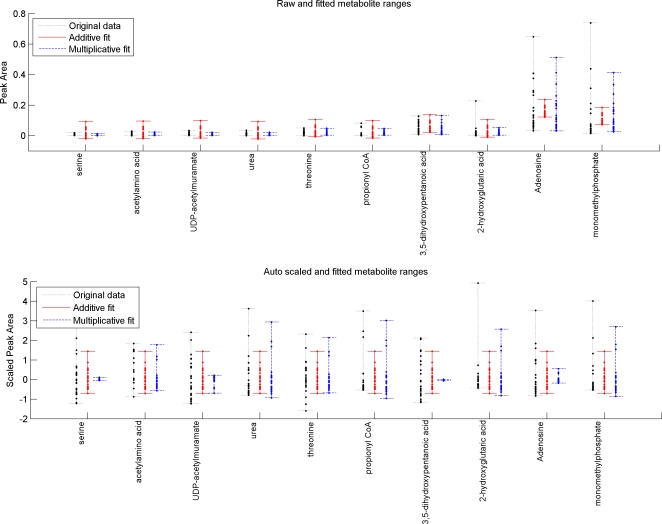
Concentration ranges for 10 metabolites before (top figure) and after (lower figure) autoscaling. Data is taken from E. Coli data as used in the remained of this paper. The whiskers indicate the total concentration range for each of the 10 metabolites. Each metabolite is represented three times. The left black lines for each metabolite are the actual concentrations. The middle red line indicates the fit/model with an additive model. The right blue lines indicate the fit/model with a blue multiplicative model.

For illustrative purposes, some metabolite measurements are plotted in Figure 2. The upper part of Figure 2 shows the concentration range of 10 metabolites (dotted black line; left) together with an additive fit (red line; middle) and the multiplicative fit (dashed blue line; right) for this set of 10 metabolites. The lower part of Figure 2 shows the same fit, but after auto scaling the data. It can be seen from Figure 2 that the range of an additive fit is the same for all metabolites and is given by the range of the α_i_'s values. It is clear that an additive model has large difficulties modeling data with highly varying ranges for the metabolites. This justifies scaling of the data. The offsets of these ranges are determined by the values of the β_j_'s. The range of a multiplicative fit can be more dynamic since it is determined by a multiplication of the values α_i_'s and β_j_'s. Additive and multiplicative simple components have clearly a different behavior.

**Figure 3:**
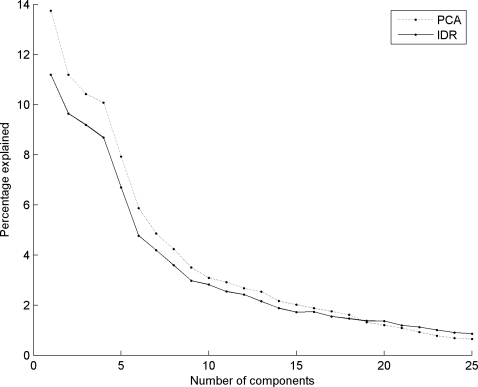
Percentage explained of original dataset given a certain number of components. Solid line represents IDR components, dotted line represents PCA components. See text for explanation.

Figure 3 shows the percentage of the original data set captured by PCA and IDR components. As expected, the PCA components explain a larger part of the data, since IDR components are constrained PCA components and thus explain less variance. IDR components >18 explain more than the original PCA components. This is easily explained, since the first 18 PCA components have almost explained the total variation in the data set, while the IDR components still capture variance that was left out by earlier IDR components. For the remainder of this paper we will focus on the first six components. They describe the largest effects in the data set and give us a clear understanding of IDR and plaid.

**Figure 4:**
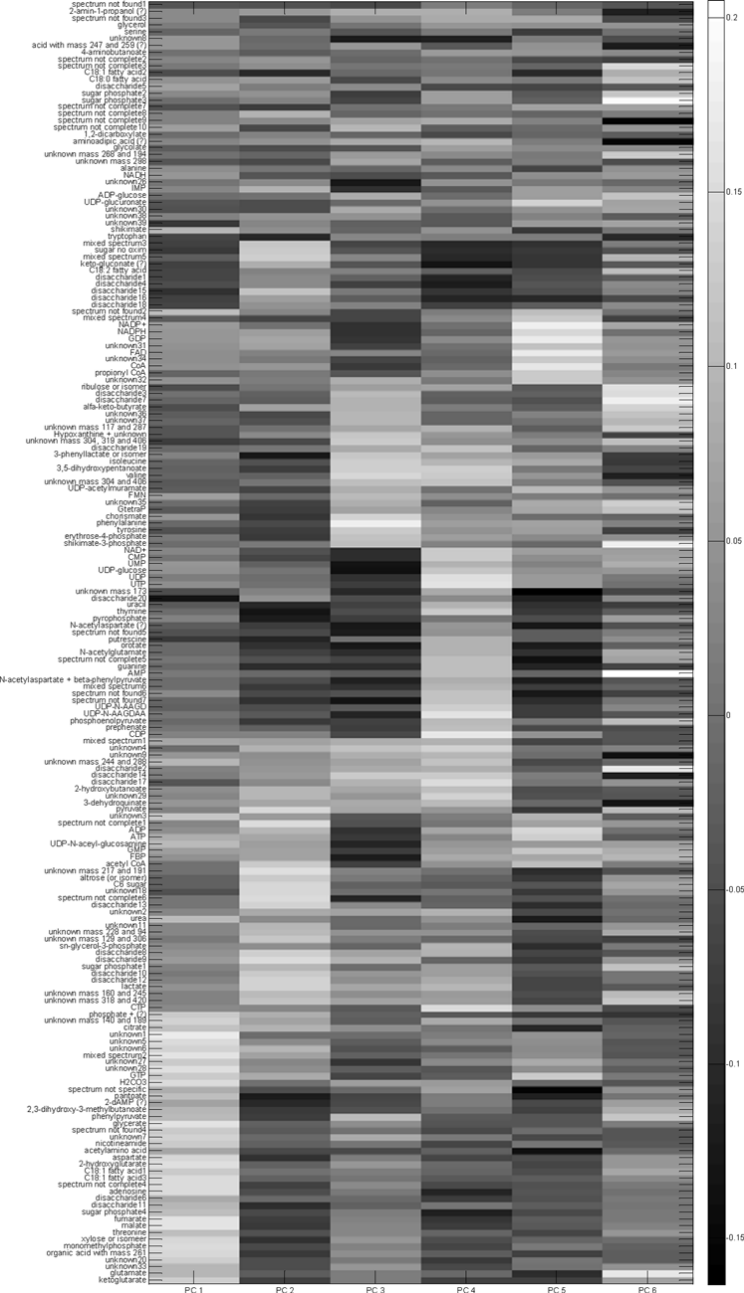
PCA solution. The values of the loadings are indicated by a grayscale color as indicated by the colorbar. The grouping of metabolites is identical to the grouping of the plaid solution for clarity.

Figure 4 shows the loadings of the PCA solution for six components in a gray-scale fashion. This figure clearly shows the problem of PCA for interpreting the solution: all components have contributions from all metabolites. This point exactly illustrates the reason for developing simplivariate models.

**Figure 5:**
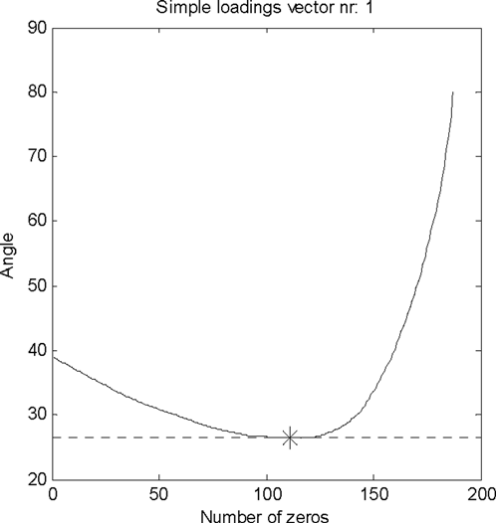
Determination of the optimal number of zeros for the first IDR component. The optimum is chosen where the angle between the simple component and principal components is minimal. This is indicated by a dotted line and an asterisk.

Figure 5 shows the determination of the optimal number of zeros in the first IDR simple component loading. The minimum is indicated by the dotted line and an asterisk. Each IDR component has a different number of zeros that results in a minimal angle between simple IDR component and original loadings. For the first IDR component, the optimal angle is 26.4 degrees and a total of 110 zeros is introduced in this simple component loading, while 78 loadings are non-zero.

**Figure 6:**
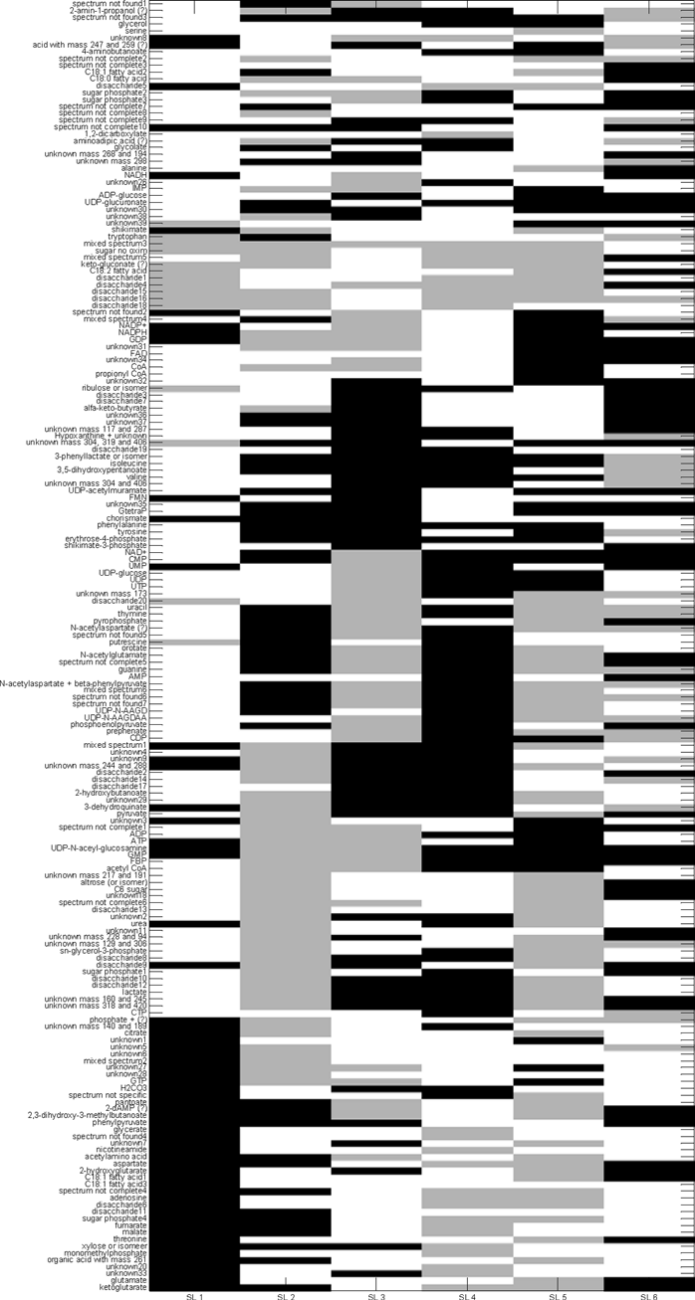
The first 6 IDR components obtained with deflation. Black squares indicate a +1, white indicates a zero, grey indicates a −1. The grouping of metabolites is identical to the grouping of the plaid solution for clarity.

**Figure 7:**
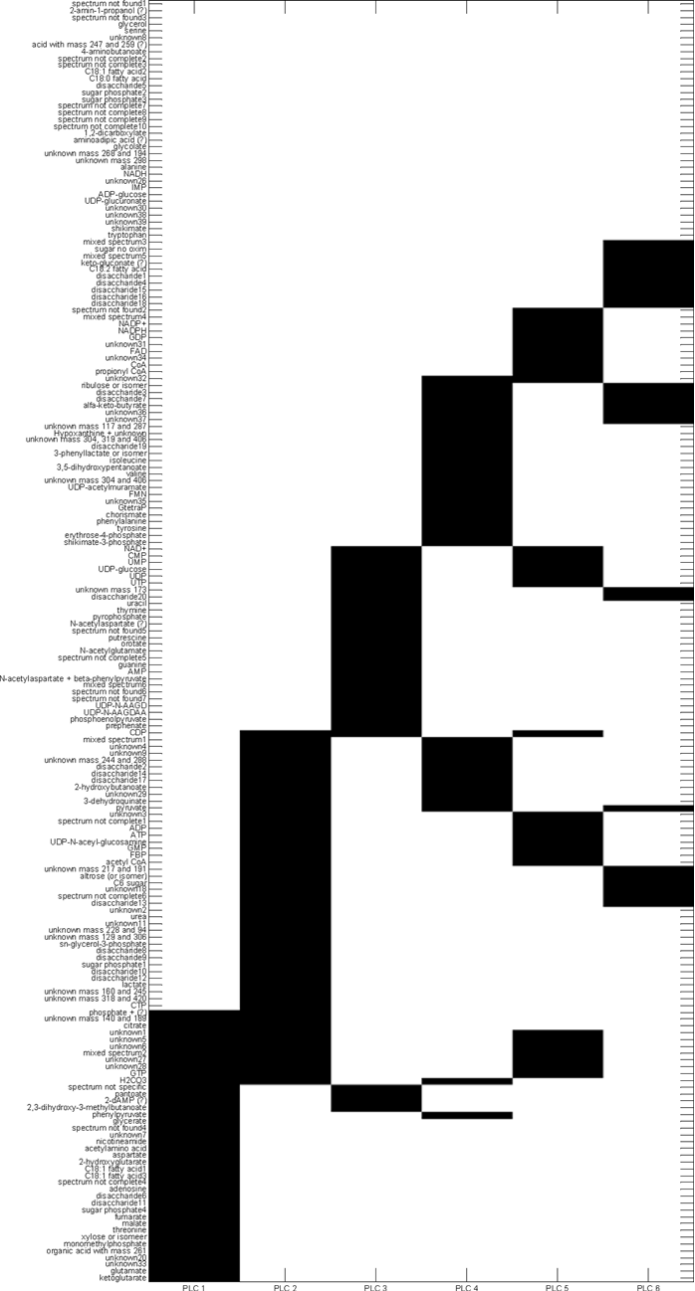
The first 6 plaid components. Black squares indicate a +1, white indicates a zero. Results have been grouped as much as possible for clarity.

Figure 6 shows the IDR simple loading vectors for the first six loadings. There is a clear distinct pattern of metabolite concentrations entering the loading (either 1 or −1, indicated by black and grey and metabolite concentrations not entering the loading (being zero, indicated by white). Figure 7 shows the first six plaid models. In Figure 4, 6 and 7 all metabolites have been ordered in such a way that metabolites are grouped as much as possible according to the different plaid clusters. Since the plaid models are only created in the variable mode (which is always the case for IDR), the object mode is not shown. One difference between plaid clusters and IDR components is striking: plaid clusters contain less metabolites and are easier to interpret. The intrinsic mechanism to lower the number of selected metabolites in IDR is automatic and cannot be intervened with. The number of zeros introduced in IDR is regulated by the optimization criterion (see step 6 of IDR algorithm) and artificially lowering the number of metabolites would yield a threshold PCA, which basically cuts of loadings values above a certain value. Hence, the interpretability can therefore not be increased. Initially, plaid also selects (too) many metabolites, however the pruning mechanism (present in the original algorithm; see Materials and Methods) is able to remove ill-fitting metabolites (see Table 1 for the settings that have been used in the plaid algorithm).

**Figure 8:**
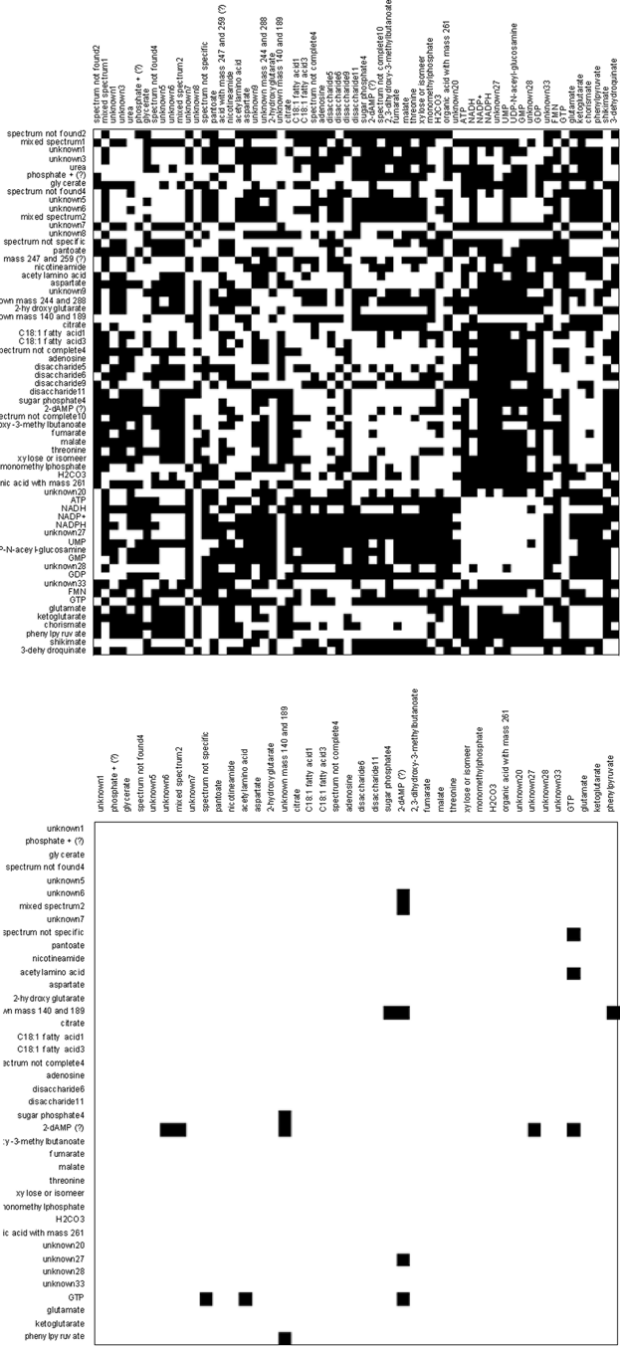
Correlations between metabolites present in IDR component 1 (top part) and plaid component 1 (bottom part). Positive correlations are indicated by a white square, negative correlations are indicated by a black square.

Although IDR and plaid have different underlying models, multiplicative or additive, there are similarities between the IDR components in Figure 6 and the plaid models in Figure 7. Many of the metabolites that are selected by IDR are also selected by the plaid models. One phenomenon is strikingly present in Figures 6 and 7. In plaid component 1, only metabolites are present that have a positive IDR value (black in Figure 7). In plaid component 2 only metabolites are present that show an IDR value of −1 in IDR component 2. Plaid components 3 and 4 are even more illustrative, since they are both represented by IDR component 3: plaid component 3 corresponds to IDR values of −1 and plaid component 4 corresponds to IDR values of +1. The reason for this phenomenon is that the additive plaid models can only represent positively correlated metabolites, missing an important part of the relationships in the data. This idea is illustrated by Figure 8 where the correlations are shown between the metabolites in IDR component 1 and between the metabolites in plaid cluster 1. What we clearly see, also in the distributions of the correlation coefficients, is that the plaid cluster contains (almost) no negatively correlated metabolites, while metabolites in IDR component 1 can be positively and negatively correlated. The differences between IDR and plaid become larger for higher components/plaid models.

### Biological interpretation

There are too many metabolites present in each IDR components to come to a meaningful analysis of the IDR results. However, the plaid clusters are relatively simple and contain biological meaningful metabolite clusters. For instance, the first plaid cluster contains all intermediates of the Krebs cycle whose concentration is above the detection limit in this data set, i.e. fumarate, malate; 2-ketoglutarate, and citrate (Fig. 7). Moreover, three metabolites which are just one enzymatic step removed from these TCA cycle intermediates, i.e. 2-hydroxyglutarate, glutamate and aspartate are also present in this first plaid cluster.

Another example is plaid cluster 4 that contains many intermediates of the phenylalanine biosynthesis pathway, i.e. erythrose-4-phosphate, 3-dehydroquinate, shikimate-3-phosphate, chorismate, phenylpyruvate, and phenylalanine itself, and several compounds which are side routes of this pathway, i.e. 3-phenyllactate, and tyrosine. Interestingly, prephenate, an intermediate at the splitting point of the phenylalanine and tyrosine biosynthesis routes, is not clustered in plaid cluster 4 but in plaid cluster 3. In contrast, when analyzing this data set by IDR, all the phenylalanine-related intermediates described above, including prephenate, end up in the same IDR component, i.e. IDR component 3 (Fig. 6). However, prephenate shows a negative loading while all other intermediates have a positive loading. One of the enzymes catalyzing the formation of prephenate (chorismate mutase encoded by pheA) is controlled by feedback inhibition by phenylalanine and also the two enzymes catalyzing its conversion (prephenate dehydratase and prephenate dehydrogenase) are controlled by feedback inhibition by phenylalanine and tyrosine, respectively. This might very well explain why this intermediate (prephenate) shows a negative correlation with the other phenylalanine intermediates (IDR analysis) and thus ends up in a different plaid cluster. Remarkably, shikimate, another phenylalanine biosynthesis intermediate, is neither clustered in plaid cluster 4 (Fig. 7) nor in IDR component 3 (Fig. 6). Interestingly, ppGpp, a major regulator of cellular metabolism, is present in plaid cluster 4/IDR component 3 indicating a link between phenylalanine biosynthesis and the stringent response in *E. coli*.

The most useful results are obtained with plaid which models (patches of) data with an additive model while IDR uses a multiplicative model. It is possible to mix both models to obtain a mixed model representation (see section on simple structures, model number 4). Mixed models might also help to further strengthen the plaid clusters. Additive plaid models can only contain positively correlated metabolite concentrations, while metabolites that are negatively correlated can still be part of the same biochemical process.

### Conclusions

The presented framework provides a good basis for simplivariate data analysis models. The two presented methods IDR and Plaid fit well in this framework. IDR suffers from too many selected metabolites which makes it rather ineffective for creating more interpretable models. This selection is intrinsic for the method and cannot be tuned. Plaid, on the other hand, was shown to be very effective in creating clusters with distinct biochemical meanings. This shows that the concept of simplivariate models is valuable.

The Plaid models also have shortcomings, notably, their inability to model metabolites belonging to the same processes having either positive or negative correlations. This can possibly be overcome by using simple components with a mixed-model structure. Moreover, the pruning mechanism present in plaid that prevents that too many metabolites are selected in a plaid cluster, remains a crude way of cleaning up a solution. It is inefficient to first create large plaid clusters (at a certain computational cost) and decreasing them after they are finished. By more carefully optimizing a plaid cluster this should be prevented. This will be subject of further research.

The framework allows for any simple component structure to include in the simplivariate model. When some of the metabolites are known to be linked in certain experiments by interlinked pathways and/or co-regulation, then these can be forced in one simple component with a structure reflecting these pathways/ this co-regulation. Also metabolic network information can be used to choose simple component structures. All these extensions are the subject of a follow-up paper.

### Notation

Matrix **X** (boldface), vector **x** (boldface), scalar *x* (italic).

Sizes: **X** (*I* objects×*J* variables), objects, *i* = 1,…,*I*; variables *j* = 1,…,*J*; groups *k* = 1,…,*K*; Each *k* represents a simple component that are used to described the data.

Group memberships: δ_jk_ = indicator for group membership of variable *j* in group *k* (δ_jk_ = 1 if variable *j* is present in group *k*, 0 otherwise); γ_ik_ = indicator for group membership of object *i* in group *k* (γ_ik_ = 1 if object *i* is present in group *k*, 0 otherwise).

PCA-scores: **T** (*I*×*R*), **t**_r_ (*r* = 1,…*R*), *t*_ir_. (*R* = number of principal components used)

PCA-loadings: **P** (*J*×*R*), **p**_r_, *p*_jr_.
